# Evaluating CSF Flow (Cine MR) and Clinical Outcomes in Patients with Chiari Type 1 Who Underwent Dural Relaxation or Durotomy

**DOI:** 10.3390/brainsci16070685

**Published:** 2026-06-29

**Authors:** Durdu Mehmet Babaoğlan, Mahmut Ferat, İsmail İştemen, Can Sezer, Mehmet Volkan Harput, Ali Arslan

**Affiliations:** Department of Neurosurgery, Adana Faculty of Medicine, University of Health Sciences, Kisla Neighborhood, Dr. Mithat Ozsan Boulevard, 4522 Street No. 28, 01230 Adana, Türkiye; mahmutferatmd@gmail.com (M.F.); drismailistemen@gmail.com (İ.İ.); mdcansezer@gmail.com (C.S.); drvolkanharput@gmail.com (M.V.H.); aliarslan26062006@hotmail.com (A.A.)

**Keywords:** Chiari malformation type I, Cine MRI, CSF flow, dural relaxation, durotomy

## Abstract

**Highlights:**

**What are the main findings?**

- Cine MRI demonstrated significant postoperative improvement in CSF flow dynamics in patients with Chiari malformation type I.
- Increased postoperative CSF flow velocities were associated with favorable clinical outcomes, while decreased flow values were observed in patients requiring reoperation.

**What are the implications of the main findings?**

- Cine MRI may serve as an objective tool for evaluating surgical adequacy and postoperative follow-up in CM-I patients.
- Dural relaxation provides effective decompression with lower CSF-related complication rates compared with durotomy.

**Abstract:**

**Background**: Cine magnetic resonance imaging (MRI) is increasingly used to evaluate abnormalities in the cerebrospinal fluid (CSF) flow in Chiari Malformation Type I (CM-I). However, its role in postoperative follow-up remains unclear. **Methods**: A retrospective analysis was performed on 58 CM-I patients who underwent foramen magnum decompression using dural relaxation or durotomy techniques. CSF flow velocities, measured using Cine MRI, were evaluated preoperatively and at one and six months postoperatively. **Results**: Postoperative CSF flow velocities were significantly increased in both follow-up periods compared to preoperative measurements (*p* < 0.001). The CSF flow velocity at six months postoperatively was also significantly higher than at one month postoperatively (*p* < 0.001). In patients with increased cerebrospinal fluid flow, the main VAS (Visual Analog Scale) score decreased and the SF-36 score increased, while growth was observed in areas where postoperative changes decreased, and these areas required reoperation. **Conclusions**: Dural relaxation or durotomy with foramen magnum decompression effectively improves CSF flow dynamics in CM-I patients. Cine MRI can provide valuable information regarding surgical adequacy and postoperative follow-up.

## 1. Introduction

Chiari malformations (CMs) comprise a spectrum of anomalies involving the hindbrain, cerebellum, brainstem, skull base, and cervical spinal cord. The pathophysiology and natural course of this complex group of disorders have not yet been fully elucidated [1,2,3].

Although Chiari malformations are classified according to the presence and degree of herniation of the posterior fossa structures (Types 1, 2, 3, and 4), additional subgroups such as Type 0 (Chiari-like syndrome), Type 1.5, Type 5, and Complex Chiari have also been described [4,5,6,7]. These variations create challenges in diagnosis and surgical decision-making.

CM Type I is the most isolated and most frequently encountered form of Chiari malformation and, although generally idiopathic, may rarely occur due to genetic or iatrogenic causes. The differentiation of this condition from Complex Chiari, which is characterized by additional craniovertebral junction anomalies, is particularly important in determining the surgical approach [8,9].

Routine radiological evaluation of CM includes MRI and CT assessment of the craniovertebral junction. Cine MRI (phase-contrast CSF flow MRI) has also become increasingly common, particularly in preoperative evaluation. Cine MRI was first introduced by Citrin et al., who demonstrated pulsatile CSF flow alterations synchronized with the cardiac cycle [10,11]. In patients with Type 1 CM, the technique was first applied by Bhadelia et al., who reported impaired preoperative CSF flow and postoperative improvement in a patient operated on for Type 1 CM [12,13].

Various surgical approaches are available for Type 1 CM, the most widely accepted of which is foramen magnum decompression (FMD); however, technical variations exist within this approach. Suboccipital craniotomy with durotomy (+duraplasty) is the best-known technique. Alternatively, dural splitting or dural relaxation, in which only the outer layer of the dura is opened, may also be preferred. Variations may also exist regarding the extent of decompression.

In this study, we evaluated whether Cine MRI findings obtained at the level of the foramen magnum in patients with Type 1 CM who underwent decompression with either durotomy or dural relaxation reflected the clinical outcome, whether the surgical decompression was sufficient, and whether Cine MRI could be incorporated into routine postoperative follow-up.

## 2. Materials and Methods

### 2.1. Study Design and Patients

**Figure 1:**
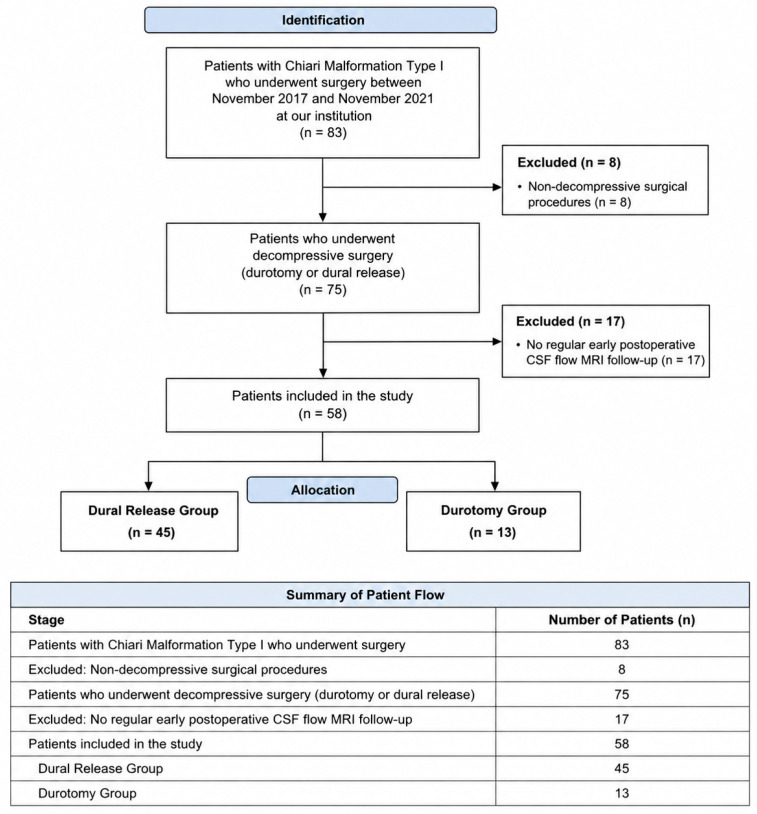
Patient selection flow diagram.

Between November 2017 and November 2021, 83 patients diagnosed with CM underwent surgery at our institution. Medical records show that 75 of these underwent decompressive procedures (durotomy or dural release). Of these, 58 patients, who were regularly monitored in the early postoperative period using CSF flow MRI, were evaluated within the scope of our study. Dural release was performed in 45 of these patients, and durotomy in 13 (Figure 1).

### 2.2. Surgical Technique

**Figure 2:**
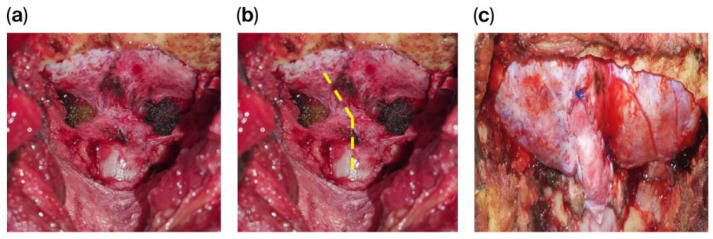
In a patient who underwent FMD and durotomy: (**a**) appearance of the dura mater after suboccipital craniotomy, (**b**) schematic illustration of the planned durotomy line (Yellow dash line: semi-Y shaped), (**c**) appearance after duraplasty.

All patients underwent surgery in a sitting position using microsurgical techniques and a fixed head retainer. Suboccipital craniotomy was performed via a midline vertical skin incision, and the posterior arch of C1 was removed in all patients. In patients with herniation of the cerebellar tonsils up to the level of C2, the posterior arch of C2 was also routinely excised. The tense horizontal fibrous band frequently encountered at the level of the foramen magnum was carefully released in all cases. In the dural release technique, after removal of the fibrous bands, only the outer layer of the dura was incised with vertical incisions, not the full thickness as in durotomy. In those who underwent durotomy, watertight duraplasty was performed with graft assistance (Figure 2).

Most of the patients included in the study (43 patients) underwent the dural release technique. Durotomy was preferred in patients with syringomyelia who had cerebellar tonsillar herniation extending to C2 (patients).

During durotomy, arachnoid adhesions were microsurgically dissected to ensure patency of the fourth ventricle. Aggressive surgical maneuvers were avoided in patients undergoing durotomy due to the risk of complications such as posterior inferior cerebellar artery (PICA) injury, brainstem injury, or arachnoiditis. None of these complications were observed in our series. All patients underwent MRI within the first 24 h postoperatively to assess the adequacy of decompression. Patients were followed up for at least 3 days postoperatively, especially for wound-related complications such as CSF leakage.

### 2.3. Radiological Evaluation

All patients underwent cervical MRI as part of the routine preoperative evaluation. Cervical CT including the craniovertebral junction was also evaluated in all patients to differentiate Type 1 CM from Complex Chiari malformation. CSF flow MRI studies, which constituted the basis of this study, were obtained preoperatively and at 1 and 6 months postoperatively.

### 2.4. Clinical Evaluation

Suboccipital headache aggravated by the Valsalva maneuver was assessed as part of the clinical evaluation. Pain severity was recorded using the VAS during the preoperative period and at the 1st and 6th postoperative months. General well-being was assessed over the specified periods using the accompanying SF-36 questionnaire.

### 2.5. Statistical Analysis

Statistical analyses were performed using IBM SPSS Statistics version 29 (IBM Corp., Armonk, NY, USA). Categorical variables are presented as numbers and percentages, whereas continuous variables are expressed as mean ± standard deviation, median, interquartile range (IQR), and minimum–maximum values.

The normality of continuous variables was assessed using the Shapiro–Wilk test. Since all variables except age did not show a normal distribution, non-parametric tests were used. Changes in CSF flow velocity and VAS score measurements over time were analyzed using the Friedman test. Effect size for the Friedman test was evaluated using Kendall’s W coefficient.

When significant differences were detected, pairwise comparisons were performed using the Wilcoxon signed-rank test. Bonferroni’s correction was applied, and *p* < 0.017 was considered statistically significant for post hoc analyses.

Postoperative change values were calculated by subtracting the preoperative CSF flow velocity from the corresponding postoperative value. Comparisons of age, pre- and postoperative CSF flow velocities, and change values according to sex were performed using the Mann–Whitney U test.

Correlations between age, CSF flow velocity measurements, and change values were analyzed using Spearman rank correlation analysis. A paired line/spaghetti plot was generated to illustrate both individual and overall temporal changes in CSF flow velocity. AI was used only in the preparation of graphics and visuals. The graph was prepared using Python 3.10 (IBM SPSS Statistics version 29 (IBM Corp., Armonk, NY, USA) in the Google Colab environment.

Two-tailed *p*-values were used throughout the analyses. Except for Bonferroni-corrected post hoc analyses, *p* < 0.05 was considered statistically significant.

## 3. Results

A total of 58 patients were included in this study, of which 32 were female (55.17%) and 26 were male (44.83%). The mean age was 30.91 ± 12.36 years (min–max: 9–70), and the median age was 29.00 years (IQR: 18.25).

CSF flow velocity values and changes according to the pre- and postoperative periods are presented in Table 1. No statistically significant differences were found between female and male patients regarding age, CSF flow velocities during different periods, or changes in CSF flow velocities (Table 2).

No correlation was detected between age and CSF flow velocities or changes in CSF flow velocity over time. Similarly, no significant correlation was found between preoperative CSF flow velocity and changes in CSF flow velocity in the first and sixth postoperative months. However, significant positive correlations were observed among CSF flow velocities measured at different time periods (Table 3).

CSF flow velocity values in the preoperative period, first and sixth postoperative months, were compared using the Friedman test. The analysis demonstrated a statistically significant difference among the three time points (χ^2^ = 83.02; df = 2; *p* < 0.001). Mean rank values were 1.08 in the preoperative period and 2.28 and 2.65 in the postoperative first and sixth postoperative months, respectively. These findings indicate that CSF flow velocity increased significantly after surgery.

Kendall’s W coefficient was calculated as 0.716, supporting a large effect size for temporal changes. Following the significant Friedman test result, post hoc pairwise comparisons were performed using the Wilcoxon signed-rank test. The significance threshold for post hoc analyses was set at *p* < 0.017 due to Bonferroni’s correction.

CSF flow velocity in the first postoperative 1 month was significantly higher than the preoperative value (Z = −6.300; *p* < 0.001). Likewise, that for the sixth month was significantly higher than the preoperative value (Z = −6.472; *p* < 0.001) and the value for the first postoperative 1 month (Z = −3.802; *p* < 0.001).

**Figure 3:**
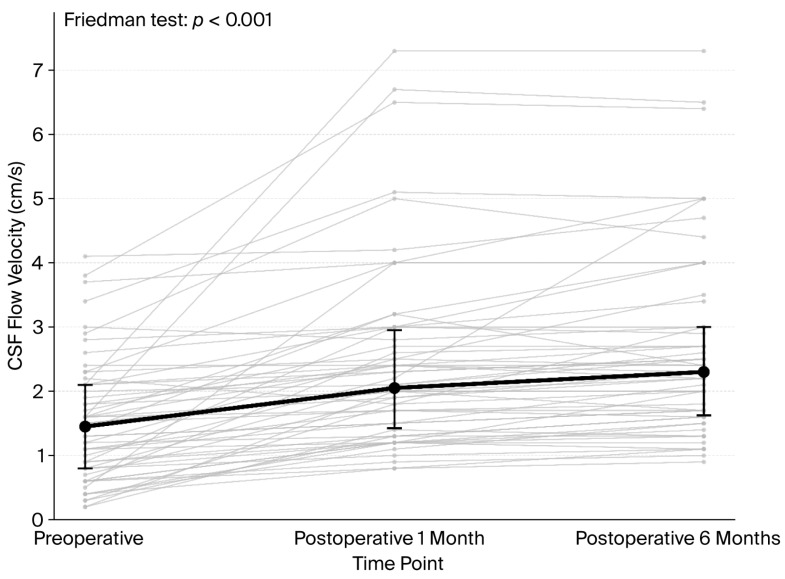
Individual changes in CSF flow velocity from the preoperative period through postoperative follow-up. Each thin gray line represents an individual patient, whereas the thick black line represents the median CSF flow velocity at each time point. Error bars indicate the interquartile range. CSF flow velocity increased significantly after surgery and remained elevated during the 6-month follow-up period. CSF: cerebrospinal fluid.

The Friedman test performed on patients’ preoperative, postoperative 1-month, and postoperative 6-month pain VAS scores demonstrated a statistically significant difference among the three time points (χ^2^ = 76.538, df = 2, *p* < 0.001; Kendall’s W = 0.660). The mean rank values were 2.89 for the preoperative period, 1.66 for the postoperative 1-month period, and 1.45 for the postoperative 6-month period. Post hoc Wilcoxon signed-rank analyses revealed that preoperative VAS scores were significantly higher than both the postoperative 1-month and postoperative 6-month scores (both *p* < 0.001). The difference between the postoperative 1-month and postoperative 6-month scores did not meet the threshold for statistical significance after Bonferroni correction (*p* = 0.047; Bonferroni-adjusted significance level *p* < 0.017) (Table 4, Figure 3)).

**Figure 4:**
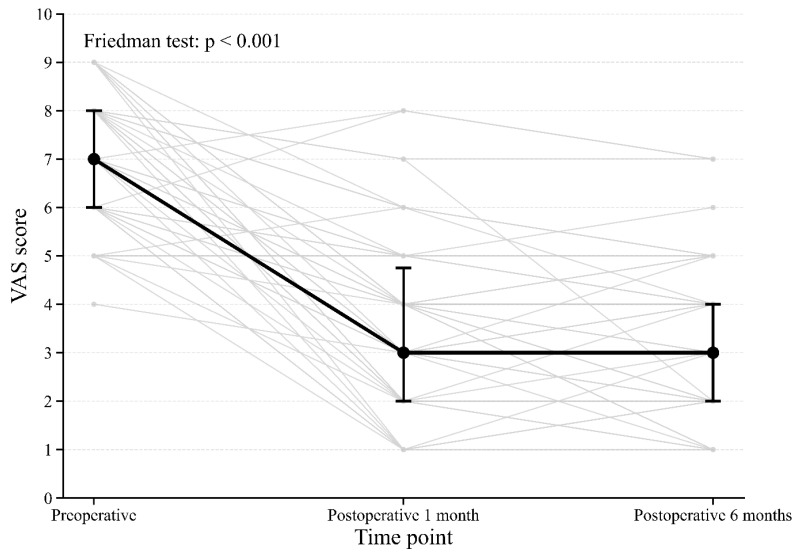
Changes in VAS pain scores over time in the preoperative period, postoperative 1st month, and postoperative 6th month. Light gray lines represent individual patient values, while the thick black line indicates the median values. Error bars represent the interquartile range (IQR). The overall difference among the time points was evaluated using the Friedman test and was found to be statistically significant (*p* < 0.001). VAS: Visual Analog Scale; IQR: interquartile range.

The patients’ preoperative, 1-month postoperative, and 6-month postoperative SF-36 scores were also evaluated. SF-36 scores showed a significant increase in postoperative follow-up compared to the preoperative period. The median SF-36 score was 38.00 in the preoperative period, rising to 74.50 at 1 month postoperatively and 78.00 at 6 months postoperatively (χ^2^ = 74.208, sd = 2, *p* < 0.001, Kendall’s W = 0.640). In Bonferroni-corrected post hoc Wilcoxon signed-rank analyses, preoperative SF-36 scores were found to be significantly lower than both the 1-month and 6-month postoperative scores (*p* < 0.001 for both). The difference between the 1-month and 6-month postoperative scores was not statistically significant (*p* = 0.126) (Table 5, Figure 4).

**Figure 5:**
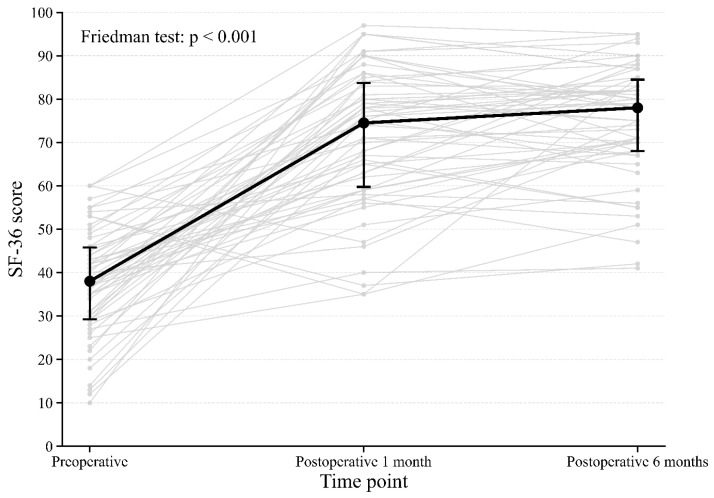
Changes in SF-36 scores over time at preoperative, 1-month postoperative, and 6-month postoperative intervals. Light gray lines represent individual values for each patient, and the thick black line represents the median value. Error bars represent interquartile ranges. The overall difference between time points was evaluated using the Friedman test and found to be statistically significant (*p* < 0.001). SF-36: Short Form-36; IQR: interquartile range.

In pairwise comparisons, compared with the preoperative period, CSF flow velocity increased in 55 patients, decreased in two patients, and remained unchanged in one patient in the first postoperative month. In the sixth postoperative month, CSF flow velocity increased in 55 patients, decreased in one patient, and remained unchanged in two patients compared with preoperative measurements. When values in the sixth postoperative month were compared with those in the first an additional increase was observed in 34 patients, a decrease in 13 patients, and no change in 11 patients (Table 6, Figure 5).

Among the patients who demonstrated a decrease in postoperative CSF flow velocity, two underwent reoperation in the first postoperative month and one in the sixth postoperative month. In these patients, postoperative VAS scores were also found to be unchanged or increased compared with the preoperative period. During the initial surgery, only one of these patients had undergone dural relaxation. At reoperation, all patients underwent durotomy and duraplasty, and the decompression boundaries were further expanded.

When CSF flow velocities were evaluated, an increase was observed between the preoperative period and the postoperative first month in all but two patients. In one of these patients, no change was detected, whereas the other patient (Patient 17) demonstrated a decrease in CSF flow velocity. In this patient, the decrease in CSF flow velocity was accompanied by an increase in the VAS score and a decrease in the SF-36 score (Table 7).

Based on the postoperative first-month evaluation, reoperation was recommended. During the initial surgery, dural relaxation had been performed; however, follow-up MRI demonstrated obstruction at the level of the foramen magnum and an extradural crowded appearance, suggesting the presence of inadequately released transverse dural bands. Durotomy was recommended according to the postoperative first-month findings, but the patient consented to the second operation only during the postoperative sixth month. The clinical and radiological findings at the postoperative sixth month were consistent with those observed at the first month.

No statistically significant differences in CSF flow velocities were observed between the postoperative first and sixth months. Similarly, VAS and SF-36 scores showed comparable findings during this period. However, in two patients (Patient 47 and 57) who demonstrated a decrease in CSF flow velocity between the postoperative first and sixth months, corresponding unfavorable clinical outcomes were observed. VAS scores remained unchanged in one patient and increased in the other. SF-36 scores were significantly reduced in these patients (Table 8).

Both patients who demonstrated negative radiological and clinical changes between the postoperative first and sixth months and were subsequently scheduled for reoperation had previously undergone durotomy. In one patient, MRI revealed a hyperintense lesion within the subdural space, consistent with dense arachnoid granulations, as well as a subdural structure causing obstruction of the spinal cord at the level of the foramen magnum.

In the other patient, a suspicious syrinx measuring approximately 0.5 mm was identified on cervical MRI obtained at the postoperative sixth month. Revision durotomy was planned in both patients, together with removal of any intraoperatively identified arachnoid bands or arachnoid granulations. In addition, decompression was extended to include the C2 lamina, which had not been removed during the initial surgery. Both patients subsequently underwent reoperation according to this surgical plan.

## 4. Discussion

The pathophysiology and associated clinical manifestations of Chiari Malformation Type I demonstrate considerable variability. Consequently, limitations remain regarding the determination of surgical indications, the selection of the optimal surgical technique, and postoperative follow-up in CM Type 1 patients.

Foramen magnum decompression (FMD) remains the most commonly preferred surgical method for CM Type 1; however, no consensus has been established, particularly concerning the extent and adequacy of decompression [14]. Significant controversy also exists regarding the preference between durotomy and dural relaxation techniques. Advocates of durotomy argue that this method is beneficial for the formation of a new cisterna magna and restoring normal CSF flow dynamics [15,16,17]. In contrast, studies have reported that dural splitting may provide similar efficacy while reducing CSF leakage and related complications. Although there are minor technical differences between these two methods, both are fundamentally based on a decompressive approach. The choice of technique largely depends on the severity of pathological findings or the surgeon’s preference. In our study, different techniques were applied according to the degree of cerebellar herniation and the presence of syringomyelia. Patient selection was therefore not randomized. The significant difference in the number of patients operated on using the two different techniques (13/45 patients) also prevented comparison of the effectiveness of these methods. This represents a limitation of our study. Such an evaluation may be possible with larger patient series and randomized patient selection.

Radiological findings are highly important in the diagnosis of CM and in determining the surgical approach, and MRI remains the most valuable modality in this regard. [18] It is particularly useful in evaluating the degree of tonsillar herniation and the adequacy of postoperative decompression. Tonsillar herniation greater than 5 mm is generally considered radiologically pathological in adults [19]. However, these radiological findings become clinically meaningful only when accompanied by clinical symptoms.

Additionally, MRI may reveal the narrowing of CSF spaces in the inferior cerebellar region or chronic compression-related changes in the cerebellar tonsils. These findings, emphasized through the experiences of Rhoton and Fessler, may facilitate diagnosis; however, they are not sufficiently objective criteria [20]. Furthermore, there are patients with negative MRI findings who are nevertheless considered suitable surgical candidates based on clinical evaluation.

Cine MRI is a valuable modality for demonstrating pathophysiological findings that cannot be identified using other radiological methods, and it has become part of routine preoperative evaluation [21,22]. In our study, this method was also assessed during postoperative follow-up. An increase in CSF flow velocity was observed to be associated with a decrease in VAS score (severity of suboccipital headache) and clinical improvement measured by the SF-36. Conversely, opposite changes in CSF flow dynamics also showed significant changes in VAS scores and SF-36 results.

In this context, Cine MRI findings, when interpreted together with clinical scales, may be useful in assessing surgical benefit and in identifying early recurrence or residual pathology following surgery.

Among the patients who underwent reoperation during follow-up, all demonstrated decreased CSF flow velocities accompanied by increased VAS scores and reduced SF-36 scores. In one patient in whom reoperation was considered based on the postoperative first-month evaluation, MRI demonstrated a restrictive appearance at the level of the foramen magnum, most likely related to persistent dural bands causing inadequate decompression and ongoing CSF flow obstruction. No patient who underwent dural relaxation required reoperation during the six-month follow-up period. In another patient, dense arachnoid adhesions were identified and considered a potential cause of secondary obstruction. Furthermore, delayed syrinx formation observed in one patient may also be interpreted as a consequence of newly developed or progressive CSF flow obstruction. In such cases, additional radiological findings may provide supportive evidence of persistent obstruction. For these two patients, further enlargement of the posterior fossa volume, including extension of decompression to the C2 lamina during reoperation, may be considered. Similarly, duraplasty with graft augmentation may provide additional benefit by increasing the decompressed surface area and improving CSF circulation.

In addition to these relatively static anatomical concepts, contemporary studies have increasingly focused on techniques capable of demonstrating the dynamic characteristics of CSF flow. The study by McIlvain et al. represents an important example of this approach. The authors found no association between the degree of cerebellar tonsillar herniation and headache severity; however, similarly to our findings, headache severity was significantly associated with Cine MRI parameters. Furthermore, CSF stroke volume measurements and Cine DENSE (Displacement Encoding with Stimulated Echoes) imaging were used to evaluate secondary neurophysiological variables related to CSF dynamics [23]. Likewise, Carlier et al. demonstrated a relationship between changes in Cine MRI findings and headache severity and reported correlations between symptom burden, additional radiological findings, and both aqueductal and cervical CSF stroke volumes [24].

Recent studies have further demonstrated that CSF dynamics should not be interpreted independently of posterior fossa morphology. D’Amico et al. showed that acquired Chiari malformation secondary to chronic CSF overdrainage may develop as a consequence of posterior fossa volume alterations and cranial remodeling. These findings emphasize that the pathophysiology of Chiari malformation involves both anatomical and hydrodynamic factors [25]. Similar to our observations, these studies suggest that CM Type I cannot be adequately characterized solely by the degree of tonsillar herniation or the presence of syringomyelia. Rather, the underlying pathology may be more accurately reflected by the dynamic CSF flow alterations demonstrated through Cine MRI. Therefore, assessment of surgical adequacy and postoperative outcomes in CM Type I should incorporate both Cine MRI findings and structural radiological changes.

From the perspective of our study, a more detailed evaluation of radiological findings, combined with contemporary methods capable of assessing CSF flow dynamics, may provide a more comprehensive understanding of the pathophysiological mechanisms underlying postoperative changes and clinical outcomes.

In our study, the outcomes of suboccipital pain exacerbated by the Valsalva maneuver were recorded using VAS, and clinical improvement was assessed using the SF-36 questionnaire. Clinical evaluation is being further refined with recently developed systems. Evaluation systems such as the Chicago Chiari Outcome Scale (CCOS) include different parameters in addition to symptoms, such as radiological findings and complications. For example, in the Chiari I Malformation Severity Scoring System defined by Holder et al., radiological findings are incorporated into the assessment alongside clinical findings [26]. More accurate results may be expected in the future with the development of new evaluation systems for CM.

In our study, the clinical course, findings, and CSF flow changes were evaluated during a postoperative follow-up period of six months. This duration may be considered short for follow-up. Nevertheless, it may help assess the early effectiveness of surgery and identify possible early recurrence or residual pathology. However, longer follow-up periods are required to determine the long-term efficacy of surgery and the value of Cine MRI in routine follow-up.

## 5. Conclusions

FMD combined with dural release or durotomy represents a safe and effective surgical approach for CM Type 1, regardless of the presence of syringomyelia. Adequate decompression facilitates CSF passage, may allow for postoperative resolution of syringomyelia if present, and can alleviate compression symptoms involving the brainstem and cervical spinal cord. In our study, the benefit of surgery in patients with CM Type 1 was evaluated using Cine MRI, and increased CSF flow velocities were observed in the group showing symptomatic improvement. In these patients, a reduction in postoperative suboccipital headache (VAS score) and clinical improvement with SF-36 were detected. Changes in CSF flow rate, supported by clinical evaluation, can help determine the effectiveness of surgery or the need for reoperation in the early postoperative period. Whether it should be included in the long-term follow-up routine can be evaluated with prospective studies supported by clinical criteria.

## Figures and Tables

**Table 1:** CSF flow velocity and changes in CSF flow velocity according to preoperative and postoperative periods.

Variable	Mean ± SD	Median (IQR)	Min–Max
Preoperative CSF flow velocity	1.51 ± 0.95	1.45 (1.33)	0.20–4.10
Postoperative 1st month CSF flow velocity	2.43 ± 1.44	2.05 (1.63)	0.80–7.30
Postoperative 6th month CSF flow velocity	2.65 ± 1.44	2.30 (1.50)	0.90–7.30
Postoperative 1st month change	0.92 ± 1.11	0.50 (1.10)	−0.40–5.20
Postoperative 6th month change	1.14 ± 1.18	0.75 (1.22)	−0.20–5.20

CSF flow velocities are presented in cm/s. Change values were calculated by subtracting the preoperative measurement from the postoperative measurement. SD: standard deviation; IQR: interquartile range.

**Table 2:** Comparison of age, CSF flow velocity, and changes in CSF flow velocity according to sex.

Variable	Female (n = 32) Median (IQR)	Mean Rank	Male (n = 26) Median (IQR)	Mean Rank	U	Z	*p*
Age (years)	30.00 (16.50)	31.89	26.50 (19.75)	26.56	339.50	−1.197	0.231
Preoperative CSF flow velocity	1.30 (1.45)	27.55	1.50 (1.28)	31.90	353.50	−0.978	0.328
Postoperative 1st month CSF flow velocity	2.15 (1.68)	30.50	2.00 (1.25)	28.27	384.00	−0.501	0.616
Postoperative 1st month change	0.85 (1.28)	32.66	0.40 (0.45)	25.62	315.00	−1.584	0.113
Postoperative 6th month CSF flow velocity	2.45 (2.23)	31.25	2.20 (1.15)	27.35	360.00	−0.876	0.381
Postoperative 6th month change	1.00 (1.38)	33.36	0.55 (0.80)	24.75	292.50	−1.934	0.053

CSF flow velocities are presented in cm/s. Change values were calculated by subtracting the preoperative measurement from the postoperative measurement. IQR: interquartile range.

**Table 3:** Correlation analyses between continuous variables.

Variables	Spearman Rho	*p*
Age–Preoperative CSF flow velocity	−0.145	0.277
Age–Postoperative 1st month CSF flow velocity	−0.106	0.428
Age–Postoperative 1st month change	0.038	0.776
Age–Postoperative 6th month CSF flow velocity	−0.140	0.296
Age–Postoperative 6th month change	0.023	0.863
Preoperative CSF flow velocity–Postoperative 1st month CSF flow velocity	0.693	**<0.001**
Preoperative CSF flow velocity–Postoperative 1st month change	−0.245	0.063
Preoperative CSF flow velocity–Postoperative 6th month CSF flow velocity	0.610	**<0.001**
Preoperative CSF flow velocity–Postoperative 6th month change	−0.255	0.053
Postoperative 1st month CSF flow velocity–Postoperative 1st month change	0.421	**<0.001**
Postoperative 1st month CSF flow velocity–Postoperative 6th month CSF flow velocity	0.931	**<0.001**
Postoperative 1st month CSF flow velocity–Postoperative 6th month change	0.378	**0.003**
Postoperative 1st month change–Postoperative 6th month CSF flow velocity	0.429	**<0.001**
Postoperative 1st month change–Postoperative 6th month change	0.878	**<0.001**
Postoperative 6th month CSF flow velocity–Postoperative 6th month change	0.520	**<0.001**

CSF flow velocities are presented in cm/s. Change values were calculated by subtracting the preoperative measurement from the postoperative measurement. Values shown in bold are statistically significant.

**Table 4:** Comparison of preoperative, postoperative 1-month, and postoperative 6-month VAS scores.

Period	VAS Score Median (IQR)	Mean Rank	χ^2^	Kendall’s W	*p* *	Post Hoc **
Preoperative	7.00 (2.00)	2.89	76.538	0.660	**<0** **.** **001**	Pre- vs. Postoperative 1st month ***p* < 0.001**
Postoperative 1st month	3.00 (3.00)	1.66				Pre- vs. Postoperative 6th month ***p* < 0.001**
Postoperative 6th month	3.00 (2.00)	1.45				Postoperative 1st vs. 6th month *p* = 0.047

VAS scores are presented as median (IQR). * Friedman test; Kendall’s W indicates the effect size for repeated measures. ** Post hoc comparisons were performed using the Wilcoxon signed-rank test. Following Bonferroni correction, the threshold for statistical significance in pairwise comparisons was set at *p* < 0.017. Bolded post hoc *p*-values indicate statistically significant differences after Bonferroni correction.

**Table 5:** Comparison of preoperative, postoperative 1-month, and postoperative 6-month SF-36 scores.

Period	SF-36 Score Median (IQR)	Mean Rank	χ^2^	Kendall’s W	*p* *	Post Hoc **
Preoperative	38.00 (17.00)	1.09	74.208	0.640	<0.001	Pre vs. Postoperative 1st month, ***p* < 0.001**
Postoperative 1st month	74.50 (25.00)	2.35				Pre vs. Postoperative 6th month, ***p* < 0.001**
Postoperative 6th month	78.00 (17.00)	2.56				Postop 1st vs. 6th month, *p* = 0.126

* Friedman test. ** Post hoc Wilcoxon signed-rank test with Bonferroni correction. A significance level of *p* < 0.017 was accepted in post hoc analyses after Bonferroni correction. SF-36: Short Form-36; IQR: interquartile range. Post hoc *p*-values in bold are significant after Bonferroni correction.

**Table 6:** Comparison of CSF flow velocities in the preoperative period and 1st and 6th postoperative months.

Period/Parameter	CSF Flow Velocity Median (IQR)	Mean Rank	χ^2^	Kendall’s W	*p* *	Post Hoc **
Preoperative	1.45 (1.33)	1.08	83.02	0.716	**<0.001**	Pre vs. Postoperative 1st Month ***p* < 0.001**
Postoperative 1st Month	2.05 (1.63)	2.28				Pre vs. Postoperative 6th Month ***p* < 0.001**
Postoperative 6th Month	2.30 (1.50)	2.65				Postoperative 1st Month vs. Postoperative 6th Month ***p* < 0.001**

CSF flow velocities are presented in cm/s. Values shown in bold are statistically significant. * Friedman test. IQR: interquartile range; Kendall’s W indicates the effect size of temporal changes in repeated measurements. ** Bonferroni-corrected post hoc Wilcoxon’s signed-rank test. According to Bonferroni’s correction, the significance threshold for three pairwise comparisons was accepted as *p* < 0.017.

**Table 7:** Parameters of a patient who underwent reoperation based on results from the postop 1st month.

Parameters (Results of 17th Patient)	Preoperative	Postop 1st Month	Postop 6th Month
CSF Flow Rate (cm/s)	3.0	2.8	3.0
VAS Score	6	8	7
SF-36 Score	53	37	42

**Table 8:** Parameters of 2 patients who underwent reoperation based on results from the postop 6th month.

	Parameter	Preoperative	Postop 1st Month	Postop 6th Month
47th patient	CSF Flow Rate	2.9	5.0	4.4
	VAS	6	5	6
	SF-36	31	90	71
57th Patient	CSF Flow Rate	1.6	3.2	2.4
	VAS	8	7	7
	SF-36	30	90	78

## Data Availability

Cerebrospinal fluid (CSF) flow velocities of all patients included in the study were recorded, measured at the foramen magnum level according to the Cine MRI results in the preoperative period, first and sixth postoperative months. This is presented as an appendix in table form. The authors have reviewed and edited the output and take full responsibility for the content of this publication.

## Abbreviations

MRI	Magnetic Resonance Imaging
CSF	Cerebrospinal Fluid
CM-I	Chiari Malformation Type I
CT	Computerized Tomography
FMD	Foramen Magnum Decompression
VAS	Visual Analog Scale
SF-36	Short Form-36
